# Efficacy and safety of Dengzhan Shengmai capsule in the treatment of chronic heart failure: a systematic review and meta-analysis

**DOI:** 10.3389/fcvm.2025.1385061

**Published:** 2025-02-21

**Authors:** Shenghua Lu, Yunfeng Yu, Sisi Dai, Yaqi Hu, Qin He, Rongzhen Liu, Jianhe Liu

**Affiliations:** ^1^ Department of Cardiovascular Medicine, The First Hospital of Hunan University of Chinese Medicine, Changsha, Hunan, China; ^2^ Branch of National Clinical Research Center for Chinese Medicine Cardiology, The First Hospital of Hunan University of Chinese Medicine, Changsha, Hunan, China

**Keywords:** Dengzhan Shengmai capsule, chronic heart failure, cardiac function, systematic review, meta-analysis

## Abstract

**Background:**

Dengzhan Shengmai Capsule (DZSMC) is a potential treatment for chronic heart failure (CHF). However, its specific benefits in the treatment of CHF remain unclear.

**Methods:**

Related randomized controlled trials were searched in eight databases up to January 28, 2025, and included studies were determined according to the inclusion criteria. Subsequently, the basic characteristics and data from these studies were recorded using Excel 2010. Outcome-related data were subsequently imported into Revman 5.3 for meta-analysis.

**Results:**

Fifteen randomized controlled trials with 1606 patients were included in this meta-analysis. The results showed that compared to conventional treatment group, DZSMC combination group significantly improved the clinical effective rate [RR = 1.21, 95% CI (1.16,1.26), *P* < 0.00001], brain natriuretic peptide (BNP) [MD = −112.60, 95%CI (−212.23, −12.96), *P* = 0.03], N-terminal pro-B-type natriuretic peptide (NT-proBNP) [MD = −88.27, 95%CI (−108.11, −68.42), *P* < 0.00001], left ventricular ejection fraction (LVEF) [MD = 6.42, 95% CI (5.57, 7.27), *P* < 0.00001], left ventricular end-diastolic diameter (LVEDD) [MD = −5.72, 95% CI (−7.56, −3.87), *P* < 0.00001], left ventricular end-systolic diameter (LVESD) [MD = −5.33, 95% CI (−7.41, −3.26), *P* < 0.00001], left ventricular end-systolic volume (LVESV) [MD = −20.71, 95% CI (−34.59, −6.82), *P* = 0.003], and 6 min walk test [MD = 51.90, 95%CI (19.08, 84.72), *P* = 0.002], while adverse events had no significant difference [RR = 0.70, 95%CI (0.42, 1.17), *P* = 0.17]. Funnel plots indicated no publication bias for BNP, NT-proBNP, LVEF, and LVESD, while potential bias was observed for other outcomes.

**Conclusion:**

DZSMC effectively improves clinical symptoms, cardiac function, and ventricular remodeling in CHF patients, with good safety, making it a potential adjuvant therapy for CHF. However, further research is needed to explore the long-term safety of DZSMC in order to enrich clinical evidence.

**Systematic Review Registration:**

https://www.crd.york.ac.uk/prospero/display_record.php?ID=CRD42024507368, PROSPERO [CRD42024507368].

## 1 Introduction

Chronic heart failure (CHF) is characterized by ventricular systolic and/or diastolic dysfunction resulting from abnormal changes in the structure and/or function of the heart. This condition manifests as weakness, dyspnea, and fluid retention due to impaired ventricular function and inadequate peripheral blood supply (1, 2). CHF represents the end stage of all kinds of heart disease and significantly contributes to decreased quality of life and increased mortality risk among patients with cardiovascular disease, posing a substantial public health challenge globally (3, 4). Epidemiologic data show that the global prevalence of CHF in adults ranges from 1% to 3%, with a positive correlation to age (5). The incidence rate of CHF in China is 275 per 100,000 person-years (287 for men and 261 for women), with approximately 3 million new cases diagnosed annually (6, 7). The 2022 AHA/ACC/HFSA Guidelines for the Management of Heart Failure recommend a quadruple regimen consisting of inhibitors of the renin angiotensin system, beta-blockers, mineralocorticoid receptor antagonists, and sodium glucose cotransporter 2 inhibitors as a basic treatment strategy for CHF (2). However, although these drugs have had beneficial effects on overall mortality, rehospitalization rates, progression of left ventricular dysfunction, and exercise tolerance in patients with CHF, there is still a problem of poorer prognosis and recurrent hospital admissions. Therefore, it is necessary to explore some safe and effective adjuvant therapy strategies to further improve the prognosis of CHF (8, 9).

According to traditional Chinese medicine theory, the pathogenesis of CHF is attributed to deficiencies in qi and yin, as well as blood stasis (10, 11). Treatment typically involves the use of remedies that nourish qi and yin and promote blood circulation (12–14). DZSMC is a kind of Chinese patent medicine made of *Erigeron breviscapus (vant.) Hand.—Mazz*, *Panax ginseng C. A. Mey*, *Ophiopogon japonicus (Thunb.) Ker-Gawl*, and *Schisandra chinensis (Turcz.) Baill*, which has the effect of nourishing qi and yin and activating blood circulation. DZSMC is primarily used to treat CHF, coronary heart disease, stroke, etc (12–14). It has been reported that the active ingredients of DZSMC can reduce blood viscosity and peripheral vascular resistance, inhibit intravascular coagulation, ameliorate microcirculation and cellular metabolism, improve myocardial function and blood supply to the heart and brain, and alleviate the damage of cardiomyocytes in the period of hypoxia (15). However, no meta-analysis has been published related to DZSMC, making the specific role of the it in the treatment of CHF is unclear. Therefore, this study used a systematic review and meta-analysis to assess the efficacy and safety of DZSMC in the treatment of CHF, aiming to provide evidence-based evidence for the clinical application of DZSMC.

## 2 Methods

This study adhered to the Preferred Reporting Items for Systematic reviews and Meta-Analyses (PRISMA). It has been registered in the PROSPERO (CRD42024507368) and updated on January 28, 2025.

### 2.1 Information sources and search strategy

A comprehensive search was conducted in English and Chinese databases, such as China National Knowledge Infrastructure (CNKI), EMBASE, PubMed, Sinomed, the Cochrane Library, Vip, Wanfang, and Web of Science, to identify all clinical studies from the date of establishment of each database to 28 January 2025. The subject terms were “Dengzhan Shengmai capsule” and “heart failure”, and the extra terms were obtained from MeSH and the Cochrane Library. And then the extra terms were combined with the subject terms to construct a complete search formula. Title/Abstract was set as the search field, and the detailed search equation was as follows: [(DengZhan Shengmai) AND (Heart Failure OR Cardiac Failure OR Heart Decompensation OR Myocardial Failure)]. No language restrictions were imposed. The search strategy was developed and implemented by SL and YY, and disagreements were resolved through consensus and discussion.

### 2.2 Inclusion and exclusion criteria

Inclusion criteria: (i) the included studies were randomized controlled trials. (ii) The included subjects were adults (≥18 years old) who met the diagnostic criteria for CHF (16). (iii) The experimental group was given DZSMC combined with conventional treatments, and the control group was treated with conventional treatments, including cardiotonic, diuretic, anticoagulation, antiplatelet aggregation, vasodilatation, etc. The execution standard of DZSMC is the *Pharmacopoeia of the People's Republic of China 2010 edition*. DZSMC is composed of *Erigerontis Herba*, *Ginseng Radix et Rhizoma*, *Schisandrae Chinensis Fructus*, and *Ophiopogonis Radix*, with each plant accounting for 56.6%, 11.3%, 11.3%, and 20.8%, respectively. The specification of each DZSMC capsule is 0.18 g, and the identification standard is that the content of Scutellarin (C_21_H_18_O_12_) and 4,5-Dicaffeoylquinic acid (C_25_H_24_O_12_) should not be less than 15.0 mg and 1.2 mg, respectively. Moreover, the qualitative and quantitative analysis conducted via ultra-high performance liquid chromatography coupled with tandem mass spectrometry (UHPLC-MS), integrated with chemometric approaches, revealed that DZSMC comprises a total of 55 compounds. These compounds are classified into four primary categories: 20 phenolic acids, 10 flavonoids, 15 saponins, and 10 lignans. For quality evaluation, nine compounds have been highlighted as potential chemical markers due to their high content and significant bioactivity. These include scutellarin (84.804–103.465 mg/g), 3,5-O-dicaffeoylquinic acid (3.415–6.038 mg/g), 4,5-O-dicaffeoylquinic acid (3.366–5.471 mg/g), ginsenoside Rb1 (0.492–1.071 mg/g), ginsenoside Re (0.169–0.420 mg/g), ginsenoside Rg1 (0.139–0.209 mg/g), ophiopogonin D (0.011–0.068 mg/g), schisandrin (0.273–0.887 mg/g), and schisandrol B (0.041–0.243 mg/g) (17). (iv) The primary efficacy outcome was the clinical effective rate, which refers to the proportion of patients with CHF related symptoms relieved to the total number. The secondary efficacy outcomes included N-terminal pro-B-type natriuretic peptide (NT-pro BNP), brain natriuretic peptide (BNP), left ventricular ejection fraction (LVEF), left ventricular end-diastolic diameter (LVEDD), left ventricular end-systolic diameter (LVESD), left ventricular end-systolic volume (LVESV), and 6-minute walk test (6-MWT). The safety efficacy outcome was the total adverse events.

Exclusion criteria: (i) the same research results were published repeatedly. (ii) The data were not available.

### 2.3 Selection process and data collection process

All articles were imported into NoteExpress 3.2.0 software. Subsequently, SL and YY independently screened articles, excluding duplicate and irrelevant articles and ultimately identifying articles that met the inclusion criteria. Then, SL and YY independently used a table created in Excel 2010 to record the basic information and research data of each included article. The basic information included author, publication year, source of participant, sample size, male ratio, average age, average disease duration, heart function classification, intervention, and treatment duration, and the research data contained all outcome measures for evaluating efficacy and safety. Finally, the two researchers cross checked the recorded data and negotiated to resolve any objections that arose during the process.

### 2.4 Risk of bias assessment

SL and YY independently used Cochrane's bias risk assessment tool to evaluate the bias risk of each included article. The assessment items included random sequence generation, allocation concealment, blinding of participants and personnel, blinding of outcome assessment, incomplete outcome data, selective reporting, and other sources of bias, with each item was classified into high-risk, low-risk, and unclear risk. Then, the two researchers cross checked the results of the bias risk assessment and negotiated to resolve any objections that arose during the process.

### 2.5 Data analysis

Firstly, the meta-analysis was performed using Revman5.3. The mean difference (MD) and 95% confidence interval (CI) were used as effect sizes for continuous variables, as well as risk ratio (RR) and 95% CI were used as effect sizes for dichotomous variables. Heterogeneity was assessed using the *I*^2^ test. When *I*^2^ < 50%, a fixed-effect model was used for analysis; when *I*^2^ ≥ 50%, a random-effects model was used. Statistical significance was set at *P* < 0.05. Secondly, the subgroup analyses based on the male ratio, average age, dosage of DZSMC, and treatment duration were used to evaluate clinical heterogeneity, sensitivity analysis based on randomization method was used to evaluate methodological heterogeneity, and sensitivity analysis based on leave-one-out method was used to identify a single source of heterogeneity and evaluate the robustness of the results. Thirdly, Funnel plots were generated using Revman5.3 to comprehensively evaluate the data for publication bias. An asymmetric scatter distribution on both sides of the funnel plot was considered to indicate a potential publication bias. Finally, GRADE 3.6 was used to evaluate the quality of evidence for the outcomes, with items including risk of bias, inconsistency, indirectness, imprecision, and publication bias.

## 3 Results

### 3.1 Study selection

**Figure 1:**
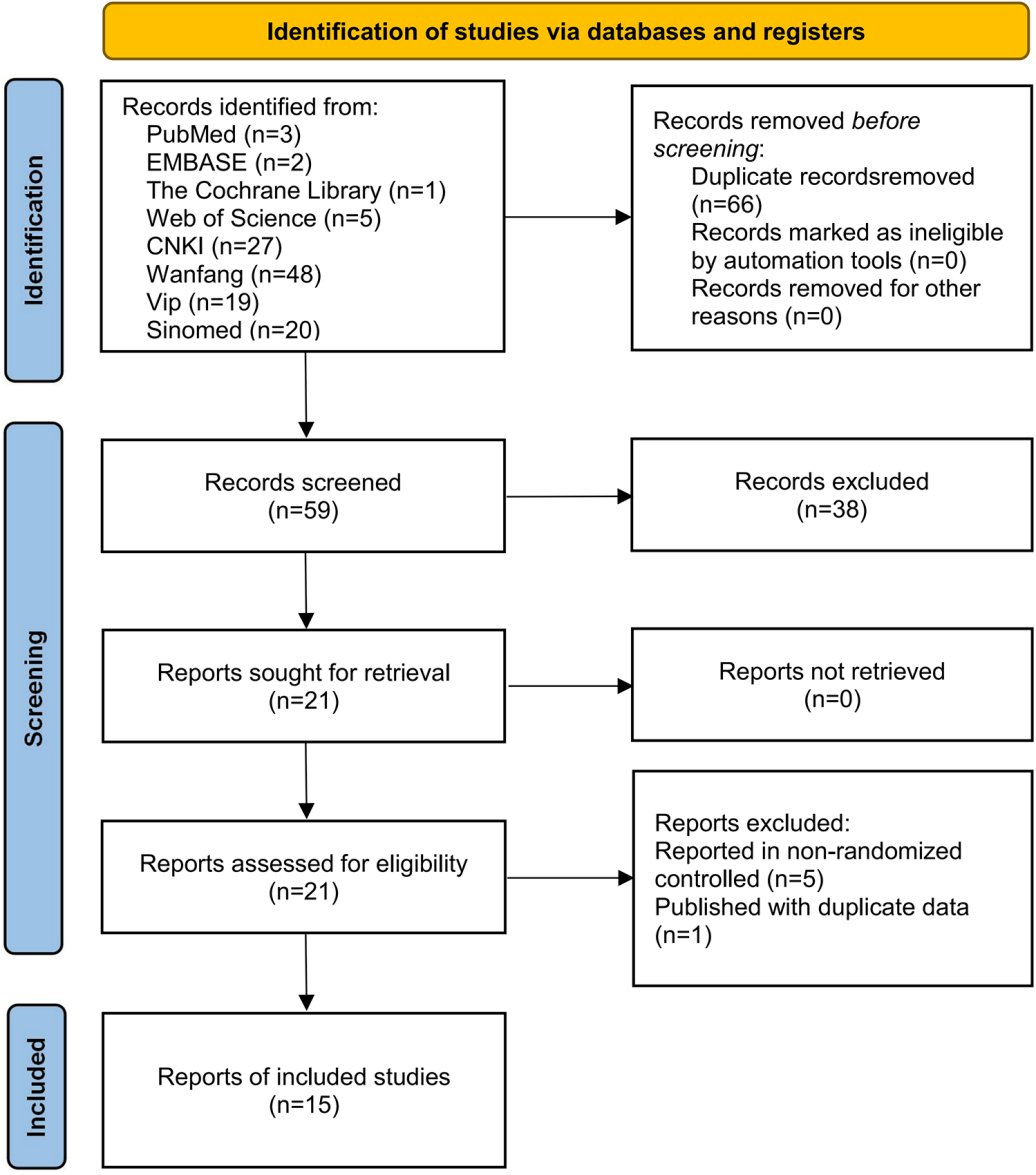
Literature screening flowchart.

A total of 125 relevant articles were obtained through a literature search, including three articles from PubMed, two from EMBASE, one from The Cochrane Library, five from Web of Science, 27 from CNKI, 48 from Wanfang, 19 from Vip, and 20 from Sinomed. Among them, 66 duplicates and 38 irrelevant articles were excluded by reading the title and abstract. Then, six irrelevant articles were excluded after reading the full text in detail for the following reasons: reported in non-randomized controlled trials (*n* = 5) and published with duplicate data (*n* = 1). Finally, 15 studies (18–32) were included for this meta-analysis. The flowchart is shown in Figure 1.

### 3.2 Study characteristics

Fifteen clinical studies (18–32) and 1,606 participants were included in this meta-analysis, spanning from 2011 to 2024. Among them, 803 participants received conventional treatment, and another 803 received DZSMC combined with conventional treatment. The male ratio of the participants was 56.1%, the average age was 62.1 years old, the average disease duration was 5.4 years, the heart function classification of participants was I–Ⅳ, and all of them from China. Additionally, the dosage of DZSMC in 14 studies (19–32) was 0.36 g each time and in one studies (18) was 0.54 g each time, and the treatment duration was between 1 week and 12 weeks. The basic characteristics of included studies are shown in Table 1.

**Table 1:** Basic characteristics of included studies.

Study	Country	Participants	Sample size	Male/%	Age/years	Disease duration/years	NYHA heart function classification	Intervention	Treatment duration/weeks
Cai (18)	China	Chinese	52	53.8	61.3	/	Ⅱ–Ⅳ	Conventional treatment DZSM Capsule 0.54 g tid	1
			52	57.7	61.4	/		Conventional treatment	1
Chen and Ye (19)	China	Chinese	75	/	/	/	Ⅱ–Ⅲ	Conventional treatment DZSM Capsule 0.36 g tid	12
			75	/	/	/		Conventional treatment	12
Hou and Zhou (20)	China	Chinese	42	59.5	65.5	5.4	Ⅱ–Ⅲ	Conventional treatment DZSM Capsule 0.36 g tid	2
			42	64.3	65.4	5.3		Conventional treatment	2
Huang et al. (21)	China	Chinese	63	58.7	65.8	/	Ⅱ–Ⅳ	Conventional treatment DZSM Capsule 0.36 g tid	4
			63	57.1	65.5	/		Conventional treatment	4
Li and Liao (22)	China	Chinese	60	58.3	68.0	/	Ⅱ–Ⅲ	Conventional treatment DZSM Capsule 0.36 g tid	4
			60	66.7	66.0	/		Conventional treatment	4
Li and Yang (23)	China	Chinese	51	62.7	53.9	2.3	Ⅱ–Ⅲ	Conventional treatment DZSM Capsule 0.36 g tid	4
			51	58.8	53.5	2.3		Conventional treatment	4
Ren et al. (24)	China	Chinese	98	49.0	63.2	6.5	Ⅱ–Ⅲ	Conventional treatment DZSM Capsule 0.36 g tid	2
			98	52.0	63.1	6.3		Conventional treatment	2
Shen et al. (25)	China	Chinese	48	58.3	56.5	5.1	Ⅱ–Ⅳ	Conventional treatment DZSM Capsule 0.36 g tid	2
			48	54.2	57.9	5.0		Conventional treatment	2
Wang (26)	China	Chinese	30	63.3	71.7	/	Ⅱ–Ⅲ	Conventional treatment DZSM Capsule 0.36 g tid	4
			30	50.0	70.6	/		Conventional treatment	4
Wu et al. (27)	China	Chinese	41	51.2	59.1	6.0	Ⅱ–Ⅳ	Conventional treatment DZSM Capsule 0.36 g tid	8
			41	46.3	58.2	5.4		Conventional treatment	8
Wu (28)	China	Chinese	46	54.3	68.5	8.2	I–Ⅱ	Conventional treatment DZSM Capsule 0.36 g tid	4
			46	60.9	69.4	7.9		Conventional treatment	4
Zhan et al. (29)	China	Chinese	45	60.0	56.8	7.2	Ⅱ–Ⅳ	Conventional treatment DZSM Capsule 0.36 g tid	8
			45	66.7	56.7	7.1		Conventional treatment	8
Zhang (30)	China	Chinese	64	54.7	66.0	2.9	Ⅱ–Ⅳ	Conventional treatment DZSM Capsule 0.36 g tid	8
			64	53.1	65.6	3.0		Conventional treatment	8
Zhang (31)	China	Chinese	43	53.5	63.3	7.9	Ⅱ–Ⅲ	Conventional treatment DZSM Capsule 0.36 g tid	2
			43	51.2	63.3	7.9		Conventional treatment	2
Zhou et al. (32)	China	Chinese	45	51.1	54.6	2.8	Ⅱ–Ⅳ	Conventional treatment DZSM Capsule 0.36 g tid	8
			45	44.4	55.9	3.2		Conventional treatment	8

DZSMC, Dengzhan Shengmai capsule.

### 3.3 Risk of bias in studies

**Figure 2:**
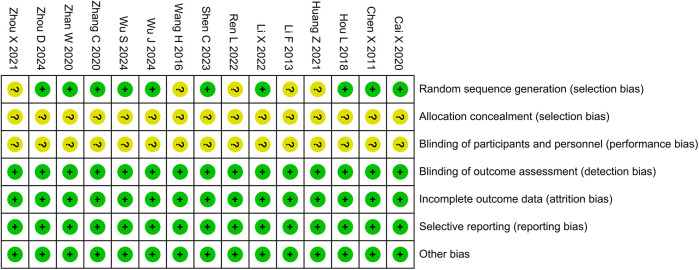
Risk of bias assessment.

Five studies were determined to have unclear risks of random sequence generation due to the lack of reporting on random methods, and 15 studies were determined to have unclear risks of allocation concealment and participant blinding due to the lack of reporting on concealment and blinding methods. Except for the above-mentioned areas, the risk of bias in all other domains was assessed as low. The bias risk assessment results are shown in Figure 2.

### 3.4 Meta-analysis

#### 3.4.1 Clinical effective rate

**Figure 3:**
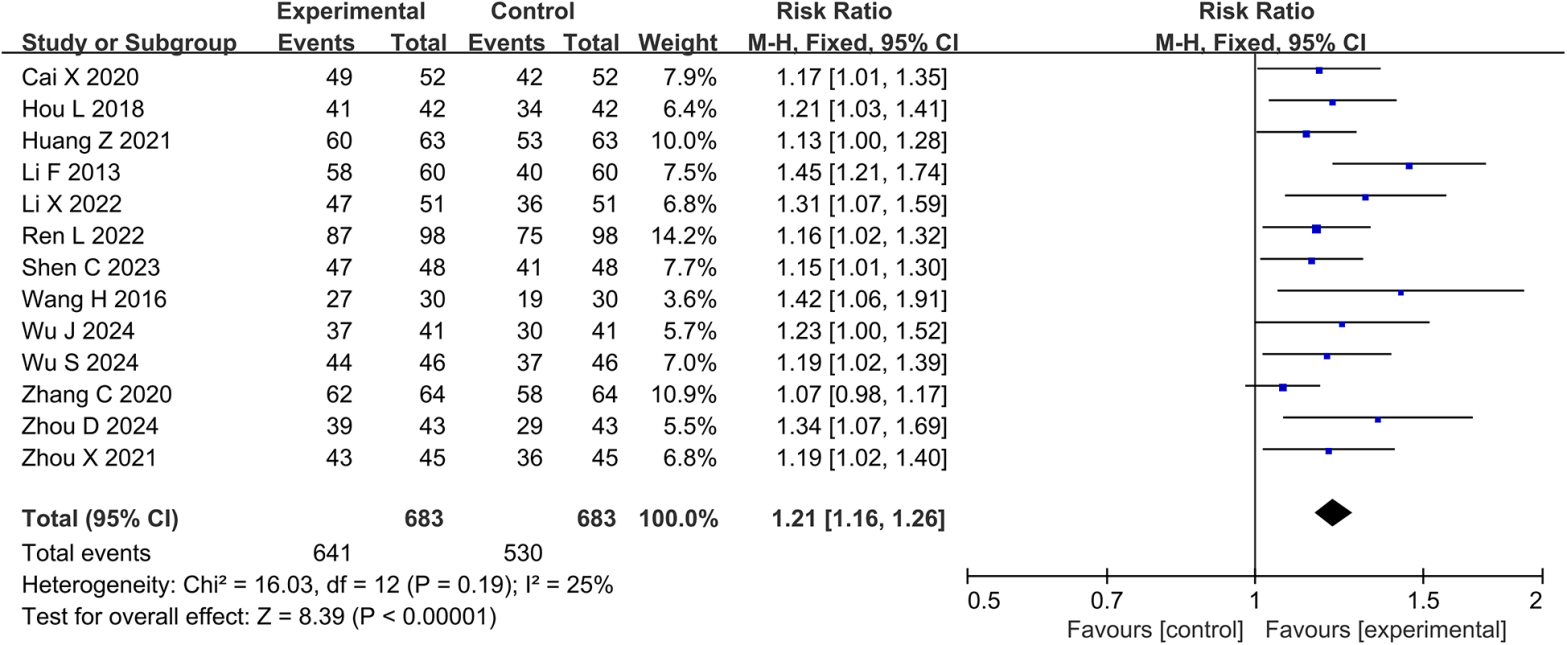
Meta-analysis results for the clinical effective rate.

Thirteen studies and 1,366 participants were included in the meta-analysis of clinical effective rate. The results showed that the DZSMC combination group significantly increased the clinical effectiveness rate by 21% compared to the conventional treatment group [RR = 1.21, 95% CI (1.16,1.26), *P* < 0.00001, *I*^2^ = 25%], as shown in Figure 3.

#### 3.4.2 BNP and NT-pro BNP

**Figure 4:**
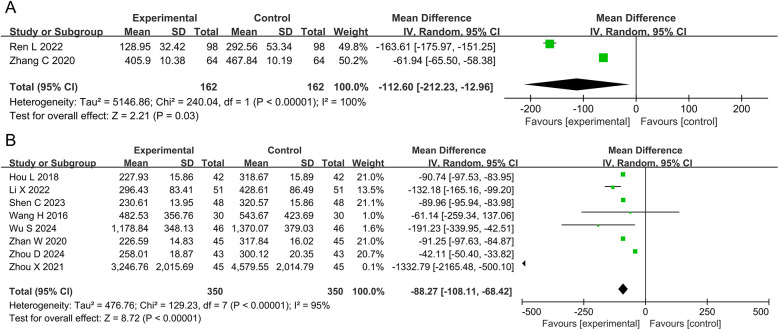
Meta-analysis results for BNP and NT-pro BNP. **(A)** BNP; **(B)** NT-pro BNP. BNP, brain natriuretic peptide; NT-pro BNP, N-terminal pro-B-type natriuretic peptide.

The meta-analyses for BNP and NT-pro BNP included two studies with 324 patients and eight studies with 700 participants, respectively. The results showed that the DZSMC combination group reduced 112.60 ng·L^−1^ BNP [MD = −112.60, 95%CI (−212.23, −12.96), *P* = 0.03, *I*^2^ = 100%] and 87.35 ng·L^−1^ NT-pro BNP [MD = −88.27, 95%CI (−108.11, −68.42), *P* < 0.00001, *I*^2^ = 95%] compared to the conventional treatment group, as shown in Figure 4.

#### 3.4.3 LVEF, LVEDD, LVESD, and LVESV

**Figure 5:**
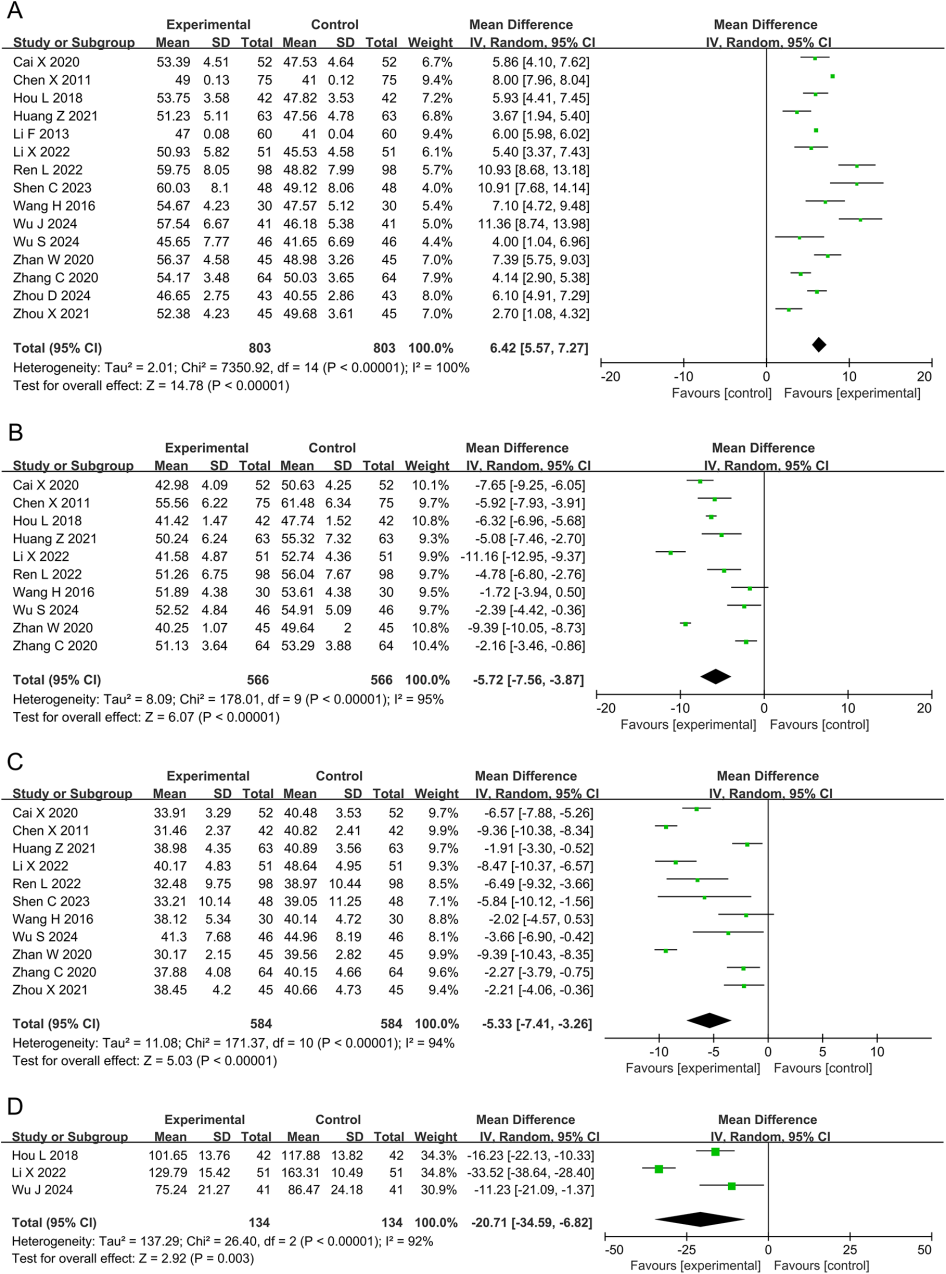
Meta-analysis results for cardiac function. **(A)** LVEF; **(B)** LVEDD; **(C)** LVESD; **(D)** LVESV. LVEF, left ventricular ejection fraction; LVEDD, left ventricular end-diastolic diameter; LVESD, left ventricular end-systolic diameter; LVESV, left ventricular end-systolic volume.

Fifteen studies and 1,606 participants were included in the meta-analysis of LVEF. The results showed that the DZSMC combination group significantly improved LVEF by 6.42% compared to the conventional treatment group [MD = 6.42, 95% CI (5.57, 7.27), *P* < 0.00001, *I*^2^ = 100%], as shown in Figure 5A.

Ten studies and 1,132 participants were included in the meta-analysis of LVEDD. The results showed that the DZSMC combination group significantly reduced LVEDD by 5.72 mm compared to the conventional treatment group [MD = −5.72, 95% CI (−7.56, −3.87), *P* < 0.00001, *I*^2^ = 95%], as shown in Figure 5B.

Eleven studies and 1,168 participants were included in the meta-analysis of LVESD. The results showed that the DZSMC combination group significantly reduced LVESD by 5.33 mm compared to the conventional treatment group [MD = −5.33, 95% CI (−7.41, −3.26), *P* < 0.00001, *I*^2^ = 94%], as shown in Figure 5C.

Three studies and 268 participants were included in the meta-analysis of LVESV. The results showed that the DZSMC combination group significantly reduced LVESV by 20.71 ml compared to the conventional treatment group [MD = −20.71, 95% CI (−34.59, −6.82), *P* = 0.003, *I*^2^ = 92%], as shown in Figure 5D.

#### 3.4.4 6-MWT

**Figure 6:**
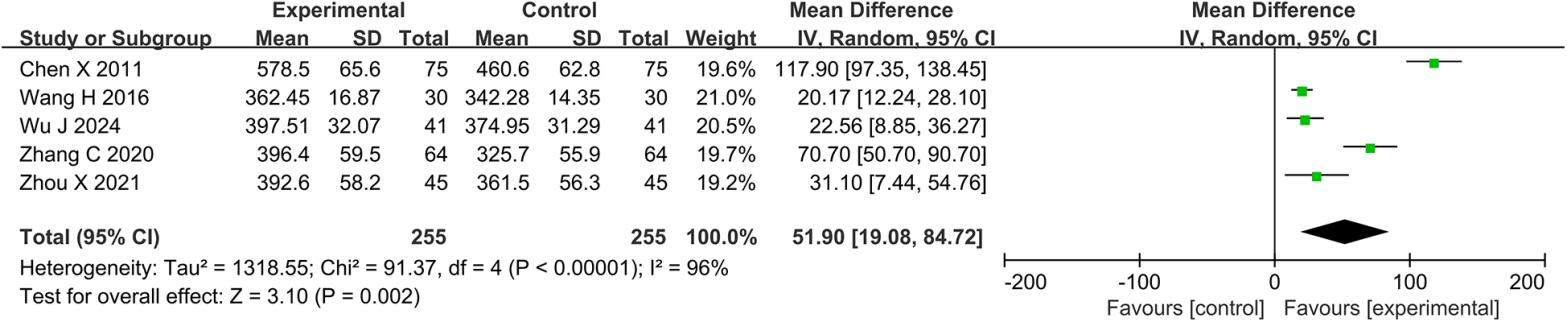
Meta-analysis results for 6-MWT. 6-MWT, 6 min walk test.

Five studies and 510 participants were included in the meta-analysis of 6-MWT. The results showed that the DZSMC combination group significantly increased 6-MWT by 51.90 m compared to the conventional treatment group [MD = 51.90, 95%CI (19.08, 84.72), *P* = 0.002, I^2^ = 96%], as shown in Figure 6.

#### 3.4.5 Adverse events

**Figure 7:**
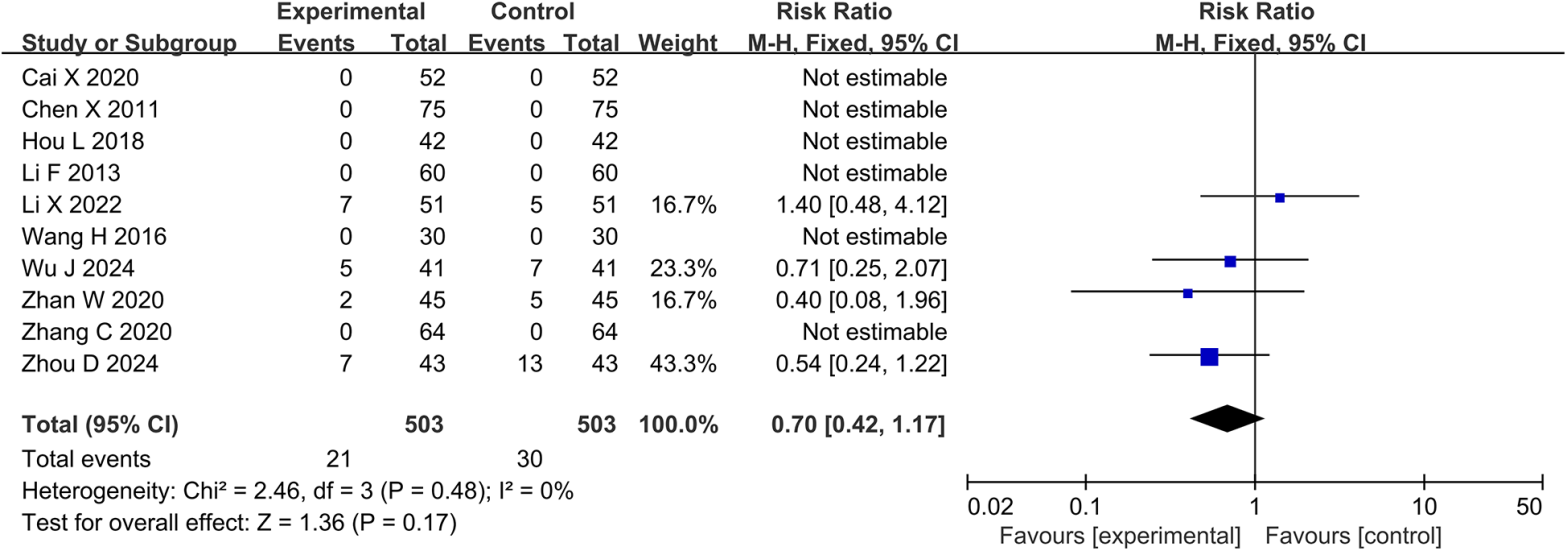
Meta-analysis results for adverse events.

Ten studies and 1,006 participants were included in the meta-analysis of adverse events. Among them, 21 patients in the DZSMC combination group experienced adverse events, including five cases of vertigo, six cases of nausea, two cases of sinus bradycardia, three cases of gastrointestinal discomfort, one case of facial flushing, one case of distension, one case of rash, one case of abdominal distension, and one case of diarrhea. 30 patients in the conventional treatment group experienced adverse events, including seven cases of vertigo, eight cases of nausea, two cases of sinus bradycardia, one case of liver function impairment, two cases of gastrointestinal discomfort, two cases of facial flushing, two cases of distension, two cases of rash, one case of abdominal pain, two cases of distension, and one case of diarrhea. The results of meta-analysis showed that there was no significant difference in adverse events between the DZSMC combination group and the conventional treatment group [RR = 0.70, 95%CI (0.42, 1.17), *P* = 0.17, I^2^ = 0%], as shown in Figure 7.

### 3.5 Subgroup analysis

Subgroup analyses explored whether the heterogeneity in BNP, NT-pro BNP, LVEF, LVEDD, LVESD, LVESV, and 6-MWT stemmed from factors such as the male ratio, average age, dosage of DZSMC, and treatment duration, as shown in Supplementary Tables S1–S4.

#### 3.5.1 Subgroup analysis based on male ratio

The subgroup analysis categorized the included studies into two groups based on the male ratio: “male ratio <60%” and “male ratio ≥60%”, as shown in Supplementary Table S1. Its results revealed that compared to the conventional treatment group, the DZSMC combination group had significant effects on NT-pro BNP, LVEF, LVEDD, LVESD and LVESV in both the “male ratio <60%” and “male ratio ≥60%” subgroups. Moreover, this subgroup analysis demonstrated that the heterogeneity in NT-pro BNP, LVEF, LVEDD, LVESD was independent of male ratio.

#### 3.5.2 Subgroup analysis based on average age

The subgroup analysis categorized the included studies into two groups based on the average age: “average age <65 years old” and “average age ≥65 years old”, as shown in Supplementary Table S2. Its results revealed that compared to the conventional treatment group, the DZSMC combination group had significant effects on BNP, NT-pro BNP, LVEF, LVEDD, LVESD, and LVESV in the “average age <65 years old” group and the “average age ≥65 years old” subgroups, while it only showed significant effects on 6-MWT in the “average age <65 years old” subgroup. Moreover, this subgroup analysis demonstrated that the heterogeneity in BNP was associated with average age, whereas heterogeneity in other outcomes was independent of average age.

#### 3.5.3 Subgroup analysis based on dosage of DZSMC

The subgroup analysis categorized the included studies into two groups based on the dosage of DZSMC: “0.36 g each time” and “0.54 g each time”, as shown in Supplementary Table S3. Its results revealed that compared to the conventional treatment group, the DZSMC combination group had significant effects on LVEF, LVEDD, and LVESD in the “0.36 g each time” and “0.54 g each time” subgroups. However, this subgroup analysis demonstrated that the heterogeneity in LVEF, LVEDD, and LVESD was independent of dosage of DZSMC.

#### 3.5.4 Subgroup analysis based on treatment duration

The subgroup analysis categorized the included studies into two groups based on the treatment duration: “treatment duration ≤4 weeks” and “treatment duration ≥8 weeks”, as shown in Supplementary Table S4. Its results revealed that compared to the conventional treatment group, the DZSMC combination group had significant effects on BNP, LVEF, LVEDD, LVESV and 6-MWT in the “treatment duration ≤4 weeks” and “treatment duration ≥8 weeks” subgroups, while significant effects on NT-pro BNP and LVESD were observed only in the “treatment duration ≤4 weeks” subgroup. Moreover, this subgroup analysis demonstrated that the heterogeneity in BNP was associated with treatment duration, whereas heterogeneity in other outcomes was independent of this factor.

In summary, the heterogeneity of BNP may be influenced by both average age and treatment duration; however, heterogeneity in other outcomes may not be related to clinical factors such as male to female ratio, average age, DZSMC dose, and course of treatment.

### 3.6 Sensitivity analysis

#### 3.6.1 Sensitivity analysis based on randomization method

We conducted sensitivity analysis on the included studies with low risk of bias in random sequence generation, as shown in Supplementary Table S5. Its results revealed that compared to the conventional treatment group, the DZSMC combination group had significant effects on BNP, NT-pro BNP, LVEF, LVEDD, LVESD, LVESV, and 6-MWT. Moreover, it demonstrated that the heterogeneity in BNP was associated with randomization method, whereas heterogeneity in other outcomes was independent of randomization method.

#### 3.6.2 Sensitivity analysis based on leave-one-out method

We conducted sensitivity analysis based on leave-one-out method to identify a single source of heterogeneity and evaluate the robustness of meta-analysis results. The leave-one-out sensitivity analysis showed no significant changes in the clinical effective rate, BNP, NT-pro BNP, LVEF, LVEDD, LVESD, LVESV, 6-MWT, and adverse events, indicating that their results were robust.

### 3.7 Publication bias

**Figure 8:**
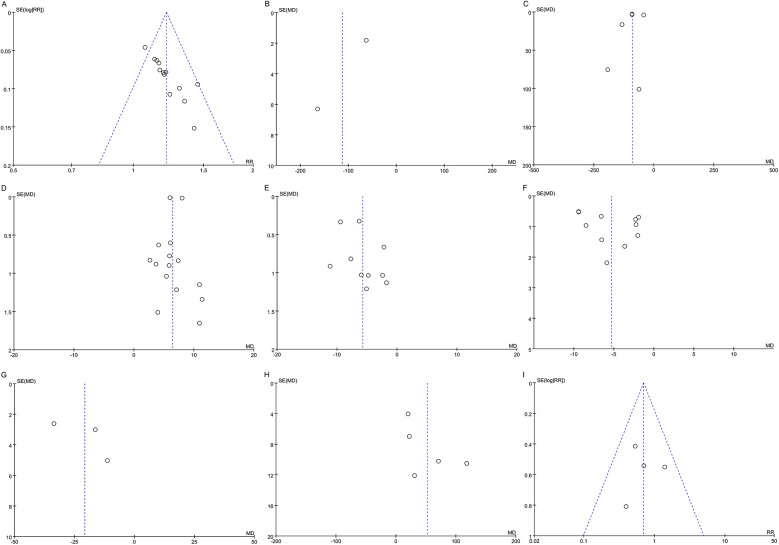
Funnel plots for publication bias. **(A)** Clinical effective rate; **(B)** BNP; **(C)** NT-pro BNP; **(D)** LVEF; **(E)** LVEDD; **(F)** LVESD; **(G)** LVESV; **(H)** 6-MWT; **(I)** Adverse events. BNP, brain natriuretic peptide; NT-pro BNP, N-terminal pro-B-type natriuretic peptide; LVEF, left ventricular ejection fraction; LVEDD, left ventricular end-diastolic diameter; LVESD, left ventricular end-systolic diameter; LVESV, left ventricular end-systolic volume; 6-MWT, 6 min walk test.

Funnel plots was used to assess publication bias for each outcome, as shown in Figure 8. Funnel plots of BNP, NT-pro BNP, LVEF, and LVESD showed symmetrical scatter distribution on both sides, indicating no publication bias. However, funnel plots of clinical effective rate, LVEDD, LVESV, 6-MWT, and adverse events showed asymmetric scatter distributions on both sides, indicating a potential publication bias.

### 3.8 Quality of evidence

The results showed that the quality of evidence for the clinical effective rate and adverse events was low, while the quality of evidence for BNP, NT-pro BNP, LVEF, LVEDD, LVESD, LVESV, and 6-MWT was very low, as shown in Table 2. Consequently, the strength of the recommendation for DZSMC is categorized as weak.

**Table 2:** Quality of evidence for outcomes.

Outcome	Risk of bias	Inconsistency	Indirectness	Imprecision	Publication bias	Quality of evidence
Clinical effective rate	Serious	None	None	None	Serious	Low
BNP	Serious	Very serious	None	Serious	None	Very low
NT-pro BNP	Serious	Very serious	None	Serious	None	Very low
LVEF	Serious	Very serious	None	None	None	Very low
LVEDD	Serious	Very serious	None	None	Serious	Very low
LVESD	Serious	Very serious	None	None	None	Very low
LVESV	Serious	Very serious	None	None	Serious	Very low
6-MWT	Serious	Very serious	None	Serious	Serious	Very low
Adverse events	Serious	None	None	None	Serious	Low

## 4 Discussion

CHF is the end stage of many heart diseases and is an important cause of reduced quality of life and death in patients with cardiovascular disease (33). Improving clinical symptoms, quality of life and reversing cardiac remodeling are key to treating CHF (34, 35). A growing number of studies have found that DZSMC enhances tolerance to cardiac hypoxia and ischemia in CHF patients, indicating its potential as a complementary strategy for the treatment of CHF (34, 35). This study included 15 randomized clinical trials involving 1,606 participants, and was the first meta-analysis of DZSMC for treating CHF, aiming to provide evidence-based evidence for its clinical application.

In terms of effectiveness, this meta-analysis indicated that DZSMC achieved benefits in outcomes such as clinical effective rate, BNP, NT-pro BNP, LVEF, LVEDD, LVESD, LVESV, and 6-MWT. First, the DZSMC combination group significantly increased the clinical effective rate by 21% compared with the conventional drug group, suggesting its effectiveness in alleviating the clinical symptoms in CHF patients. Second, the DZSMC combination group reduced BNP by 112.60 ng·L^−1^ and NT-pro BNP by 88.27 ng·L^−1^. It suggests that DZSMC reduce the risks of CHF progression, as NT-pro BNP is also an important indicator of overall prognostic efficacy in heart failure (36). Third, the DZSMC combination group improved LVEF by 6.42%, LVEDD by 5.72 mm, LVESD by 5.33 mm, and LVESV by 20.71 ml. LVEF is the ratio of stroke volume to left ventricular end-diastolic volume, while LVESD, LVEDD, and LVESV are key indicators for assessing left ventricular systolic function and ventricular remodeling, commonly used to reflect myocardial contractility and structural changes in the ventricle (37, 38). The improvement in these four outcomes suggests that DZSMC improves cardiac function and reverse ventricular remodeling to some extent. Moreover, the DZSMC combination group also significantly increased 6-MWT by 51.90 m compared to the conventional treatment group. It means that DZSMC improves the cardiac function in CHF patients, as 6-MWT is one of the more common tests used clinically to evaluate cardiac function and therapeutic efficacy in CHF patients. In summary, this meta-analysis indicates that DZSMC effectively improves clinical symptoms, cardiac function, and ventricular remodeling in CHF patients, supporting the findings of previous clinical studies.

In terms of safety, the adverse event rate was 4.17% (21/503) in the DZSMC combination group and 5.96% (30/503) in the conventional treatment group, which was comparable between the two groups, suggesting a better safety profile for DZSMC. In fact, the adverse events that occurred in both groups were mainly gastrointestinal, and were closely related to conventional treatment. Therefore, based on this meta-analysis and previous clinical reports, we believe that DZSMC is a safe therapeutic option. However, the long-term safety of DZSMC still needs to be explored in more studies, as the safety data is limited.

Subgroup and sensitivity analyses were conducted to evaluate the impact of clinical and methodological heterogeneity on the results. These analyses indicated that the heterogeneity in NT-pro BNP, LVESD, and LVESV may be attributed to average age, and the heterogeneity of BNP may be attributed to average age, treatment duration, and randomization method. Moreover, although subgroup and sensitivity analyses did not find the sources of heterogeneity for LVEF, LVEDD, and 6-MWT, the leave-one-out sensitivity analysis indicated that the meta-analysis results for each outcome were robust.

According to traditional Chinese medicine theory, CHF is caused by deficiencies in qi and yin, along with blood stasis, leading to long-term overload of the heart. Therefore, Chinese medicine experts advocate for the use of drugs that nourish qi and yin and promote blood circulation to treat CHF. DZSMC is a Chinese patent medicine made from *Erigeron breviscapus (vant.) Hand.—Mazz* as the main drug, supplemented by *Panax ginseng C. A. Mey*, *Ophiopogon japonicus (Thunb.) Ker-Gawl*, and *Schisandra chinensis (Turcz.) Baill*, which has the effects of benefiting qi and yin and removing blood stasis. Pharmacological studies have shown that the main active ingredients of *Erigeron breviscapus (vant.) Hand.—Mazz* are dicaffeinated quinic acid and ketone compounds, such as breviscapine, apigenin, hypericin, etc (39). These components can inhibit platelet and red blood cell aggregation, reduce blood viscosity, and attenuate myocardial oxidative stress injury, thereby improving the clinical symptoms of CHF (40). Moreover, experimental studies have confirmed that Shengmaisan, a Chinese patent medicine comprising *Panax ginseng C. A. Mey*, *Ophiopogon japonicus (Thunb.) Ker-Gawl*, and *Schisandra chinensis (Turcz.) Baill*, exerts a positive inotropic effect. This effect enhances vascular endothelial function, reduces myocardial oxygen consumption, and suppresses myocardial hypertrophy and fibrosis, ultimately leading to improved cardiac function and reversal of ventricular remodeling (41).

Despite adhering to the PRISMA, this study has some limitations. First, the potential for selective bias and implementation bias within the included studies is unclear, potentially compromising its overall credibility. Second, various diagnostic criteria were employed in the included studies, such as those outlined in the 2014 Guidelines for the Diagnosis and Treatment of Heart Failure in China as well as 2002 Recommendations for the Treatment of Chronic Systolic Heart Failure, among others. This diversity in diagnostic criteria introduces methodological heterogeneity that could impact the meta-analysis results. Third, treatment durations in the studies ranged from 1 week to 12 weeks, and there is a notable absence of long-term follow-up data for the efficacy of DZSMC in treating CHF. Finally, DZSMC is a commonly used Chinese patent medicine primarily within China, limiting the geographical scope of related clinical trials. Consequently, the applicability of study results may be restricted to certain ethnicities, raising questions about its efficacy in other racial groups. Multicenter, double-blind, stratified randomized controlled trials are recommended for future research to delve deeper into the impact of factors like age, disease duration, race, and treatment duration on DZSMC efficacy. Such studies can enhance the quality of evidence available for evidence-based medical research on CHF treatment with DZSMC.

## 5 Conclusion

DZSMC effectively improves clinical symptoms, cardiac function, and ventricular remodeling in CHF patients, with good safety, making it a potential adjuvant therapy for CHF. However, further research is necessary to explore the long-term safety of DZSMC in order to enrich clinical evidence.

## Data Availability

The original contributions presented in the study are included in the article/Supplementary Materials, further inquiries can be directed to the corresponding author.
