# Supplementary material for: Efficacy and safety of Dengzhan Shengmai capsule in the treatment of chronic heart failure: a systematic review and meta-analysis
Source: Front Cardiovasc Med. 2025 Feb 21;12:1385061. doi: 10.3389/fcvm.2025.1385061 (PMC11885271; doi:10.3389/fcvm.2025.1385061)
Supplement: Supplementary file 1 [file Datasheet1.pdf]

Supplementary Table S1 Subgroup analysis based on male ratio.

Outcome	Subgroup	Number of studies	I^2/%	MD (95%CI)	*P* value
NT-pro BNP	Male ratio < 60%	5	96	-79.42 (-125.15, -33.68）	0.0007
	Male ratio ≥ 60%	3	66	-94.09 (-103.77, -84.42)	< 0.00001
LVEF	Male ratio < 60%	10	88	6.51 (4.79, 8.23)	< 0.00001
	Male ratio ≥ 60%	4	3	6.01 (5.81, 6.22)	< 0.00001
LVEDD	Male ratio < 60%	8	83	-4.11 (-5.76, -2.46)	< 0.00001
	Male ratio ≥ 60%	3	96	-8.86 (-11.46, -6.26)	< 0.00001
LVESD	Male ratio < 60%	8	81	-3.73 (-5.38, -2.07)	< 0.00001
	Male ratio ≥ 60%	3	0	-9.26 (-9.94, -8.58)	< 0.00001
LVESV	Male ratio < 60%	1	0	-11.23 (-21.09, -1.37)	0.03
	Male ratio ≥ 60%	2	95	-24.94 (-41.88, -8.00)	0.004

Supplementary Table S2 Subgroup analysis based on average age.

Outcome	Subgroup	Number of studies	I^2/%	MD (95%CI)	*P* value
BNP	Average age < 65 y	1	0	-163.61 (-175.97, -151.25)	< 0.00001
	Average age ≥ 65 y	1	0	-61.94 (-65.50, -58.38)	< 0.00001
NT-pro BNP	Average age < 65 y	5	97	-87.00 (-113.80, -60.20)	< 0.00001
	Average age ≥ 65 y	3	0	-90.91 (-97.69, -84.14)	< 0.00001
LVEF	Average age < 65 y	8	88	7.39 (5.46, 9.33)	< 0.00001
	Average age ≥ 65 y	6	73	5.21 (4.18, 6.24)	< 0.00001
LVEDD	Average age < 65 y	6	93	-7.13 (-9.55, -4.71)	< 0.00001
	Average age ≥ 65 y	5	92	-3.59 (-5.92, -1.25)	0.003
LVESD	Average age < 65 y	6	90	-6.58 (-8.86, -4.29)	< 0.00001
	Average age ≥ 65 y	5	96	-3.89 (-7.68, -0.09)	0.0009
LVESV	Average age < 65 y	2	94	-22.79 (-44.62, -0.96)	0.04
	Average age ≥ 65 y	1	0	-16.23 (-22.13, -10.33)	< 0.00001
6-MWT	Average age < 65 y	2	0	24.71 (12.84, 36.57)	< 0.0001
	Average age ≥ 65 y	2	95	44.57 (-4.92, 94.06)	0.08

Supplementary Table S3 Subgroup analysis based on dosage of DZSMC.

Outcome	Subgroup	Number of studies	I^2/%	MD (95%CI)	*P* value
LVEF	0.36 g each time	14	100	6.46 (5.58, 7.34)	< 0.00001
	0.54 g each time	1	0	5.86 (4.10, 7.62)	< 0.00001
LVEDD	0.36 g each time	11	95	-5.34 (-7.19, -3.50)	< 0.00001
	0.54 g each time	1	0	-7.65 (-9.25, -6.05)	< 0.00001
LVESD	0.36 g each time	10	95	-5.19 (-7.56, -2.83)	< 0.0001
	0.54 g each time	1	0	-6.57 (-7.88, -5.26)	< 0.00001

Supplementary Table S4 Subgroup analysis based on treatment duration.

Outcome	Subgroup	Number of studies	I^2/%	MD (95%CI)	*P* value
BNP	Treatment duration ≤ 4 w	1	0	-163.61 (-175.97, -151.25)	<0.00001
	Treatment duration ≥ 8 w	1	0	-61.94 (-65.50, -58.38)	<0.00001
NT-pro BNP	Treatment duration ≤ 4 w	6	96	-87.92 (-113.61, -62.22)	<0.00001
	Treatment duration ≥ 8 w	2	88	-639.33 (-1847.65, 568.98)	0.30
LVEF	Treatment duration ≤ 4 w	10	76	6.35 (5.40, 7.29)	<0.00001
	Treatment duration ≥ 8 w	5	95	6.60 (4.20, 9.00)	<0.00001
LVEDD	Treatment duration ≤ 4 w	8	89	-5.79 (-7.61, -3.98)	<0.00001
	Treatment duration ≥ 8 w	4	98	-5.07 (-9.29, -0.86)	0.02
LVESD	Treatment duration ≤ 4 w	8	92	-5.60 (-7.97, -3.24)	<0.00001
	Treatment duration ≥ 8 w	3	98	-4.66 (-9.91, -0.59)	0.08
LVESV	Treatment duration ≤ 4 w	2	95	-24.94 (-41.88, -8.00)	0.004
	Treatment duration ≥ 8 w	1	0	-11.23 (-21.09, -1.37)	0.03
6-MWT	Treatment duration ≤ 4 w	1	0	20.17 (12.24, 28.10)	<0.00001
	Treatment duration ≥ 8 w	4	95	60.40 (16.71, 104.10)	0.007

Supplementary Table S5 Sensitivity analysis based on randomization method.

Outcome	Number of studies	I^2/%	MD (95%CI)	*P* value
BNP	1	0	-61.94 (-65.50, -58.38)	< 0.00001
NT-pro BNP	6	96	-87.62 (-107.07, -68.17)	< 0.00001
LVEF	10	89	6.76 (5.51, 8.00)	< 0.00001
LVEDD	7	96	-6.45 (-8.62, -4.29)	< 0.00001
LVESD	7	92	-6.66 (-8.83, -4.50)	< 0.00001
LVESV	3	92	-20.71 (-34.59, -6.82)	0.003
6-MWT	3	97	69.99 (13.47, 126.52)	0.02
